# In Vivo Evaluation of the Effects of SMILE with Different Amounts of Stromal Ablation on Corneal Biomechanics by Optical Coherence Elastography

**DOI:** 10.3390/diagnostics13010030

**Published:** 2022-12-22

**Authors:** Yirui Zhu, Yanzhi Zhao, Yubao Zhang, Hongwei Yang, Jiulin Shi, Hongling Cai, Dong Zhang, Guofu Huang, Xingdao He, Xiaoshan Wu

**Affiliations:** 1 School of Physics, Nanjing University, Nanjing 210093, China; 2 School of Testing and Optoelectronic Engineering, Nanchang Hangkong University, Nanchang 330063, China; 3 School of Medical, Nanchang University, Nanchang 330031, China

**Keywords:** optical coherence tomography, optical coherence elastography, biomechanical properties of corneal, corneal refractive surgery, small incision lenticule extraction

## Abstract

This work aims to depth-resolved quantitatively analyze the effect of different stromal ablation amounts on the corneal biomechanical properties during small incision lenticule extraction (SMILE) using optical coherence elastography (OCE). A 4.5-MHz ultrasonic transducer was used to excite elastic waves in the corneal tissue. The OCE system combined with the antisymmetric Lamb wave model was employed to achieve a high-resolution, high-sensitivity, and depth-resolved quantitative detection of the corneal Young’s modulus. Eighteen rabbits were randomly divided into three groups; each group had six rabbits. The first and second groups underwent -3D and -6D SMILE surgeries, and the third group was the control group, respectively. Young’s modulus of the corneal cap and residual stromal bed (RSB) were both increased after SMILE, which shared the stress under intraocular pressure (IOP). Furthermore, the Young’s modulus of both the corneal cap and RSB after 3D SMILE group were significantly lower than that in the -6D group, which indicated that the increases in the post-operative corneal Young’s modulus were positively correlated with the amount of stromal ablation. The OCE system for quantitative spatial characterization of corneal biomechanical properties can provide useful information on the extent of safe ablation for SMILE procedures.

## 1. Introduction

Small incision lenticule extraction (SMILE) is an innovative form of keratorefractive surgery. The femtosecond laser is utilized to make an intrastromal lenticule in the corneal stromal layer that is then mechanically removed via a lateral incision of 2–3 mm. The cornea is a transparent, nonlinear, elastic tissue; thus, removing the corneal tissue ultimately reduces the cornea’s biomechanical strength while increasing the refractive power [1,2]. When the biomechanical strength of the cornea is smaller than the threshold required to maintain its shape, latrogenic keratectasia occurs [3,4]. The primary concern of refractive surgeons is maintaining the safety of surgery for more patients. Corneal biomechanical parameters may be used to guide surgical decision-making. SMILE post-operative corneal cap maintains the majority of the anterior stroma compared to flap-based surgeries. In theory, SMILE has the advantage of preserving biomechanical properties; thus, it can be used to correct higher refractive errors [5,6,7]. However, many clinical studies on the dynamic response of the cornea to airflow compression after the refractive surgery have not proven significant differences between the cap- and flap-based procedures [8,9,10,11,12]. This result could be owing to the fact that current clinical devices can only detect the biomechanical properties of the cornea as a whole. In addition, they are influenced by confounding factors, such as corneal thickness and intraocular pressure (IOP). Consequently, it is critical to developing clinical devices with high spatial resolution and high detection sensitivity to reduce the influence of confounding factors on the corneal biomechanical properties, because the biomechanical properties of corneal flaps and residual stromal beds (RSBs) can be accurately quantified after the refractive surgery [6,13].

At present, elastography based on ultrasonic imaging and optical imaging approaches has been developed and applied to characterize and measure the biomechanical properties of biological tissues. The waves-based ultrasound elastography has been widely used to assess the biomechanical properties of the ocular tissues. Runze Li et at. proposed a high-resolution ultrasound elastography system to assess the biomechanics of whole ocular tissue [14]. Xuejun Qian et al. evaluated the biomechanics of the optical nerve head using a high-frequency ultrasound elastography system combined with shaker excitation [15]. However, the resolution of ultrasound imaging techniques does not allow for the stratified quantification of corneal elastic modulus. For the optical imaging methods, both Brillouin optical microscopy and optical coherence elastography (OCE) were used to detect and assess corneal biomechanics. Similar to ultrasound spectroscopy, according to the relationship between the viscoelastic properties and inherently hypersonic acoustic waves of the tissue, Brillouin optical microscopy measures the viscoelastic properties by detecting the spectral shift of the Brillouin light-scattering [16,17,18]. The Brillouin microscopy has been used to measure the biomechanical properties of the keratoconus of the human eye; the results have shown that biomechanics of the keratoconus significantly differ from those of the normal cornea [19,20]. As for the refractive surgery, Randleman et al. used porcine eyes and demonstrated that the flap-based laser-assisted in situ keratomileusis (LASIK) procedure substantially decreases the Brillouin shift in the anterior third of the stroma [21]. However, some limitations of Brillouin optical microscopy need to be considered. The refractive index and material density of the tissue are the key parameters that must be determined to accurately calculate the longitudinal modulus in the Brillouin experiments [22]. For anisotropic biology tissues, the local refractive index and density measurement increase the difficulty of in situ detection [23]. Another disadvantage of Brillouin microscopy is its long signal acquisition time owing to its low signal to noise, which limits the clinical application of Brillouin microscopy in vivo [24].

The OCE technology was introduced by Joseph M. Schmitt in 1998; this technology has been quickly developed as a noninvasive and quantitative biomechanical imaging technique owing to the optical coherence tomography (OCT), high spatial resolution, high imaging speed, and free label [25,26,27,28,29,30,31]. The OCE technique can quantify changes in the biomechanical properties of the cornea before and after cross-linking (CXL), which is a conventional treatment for keratoconus. Some studies have reported that CXL effectively increases corneal stiffness; thus, the OCE technique could be used to detect ocular diseases, such as keratoconus, in clinical applications [32,33,34,35]. Combined with an external exciter, such as the acoustic radiation force or air puff, OCE has been used to detect and analyze the effects of the corneal thickness, corneal curvature, IOP, and hydration on the biomechanical properties of the cornea [36,37,38]. Recently, Lan et al. used OCE in healthy human subjects and analyzed the effects of the respiratory and cardiac-induced eye motions noise on the corneal biomechanics measurement [39]. Zvietcovich et al. presented a novel technology that is a reverberant three-dimensional OCE; this technology helps characterize each layer of the cornea with high lateral and axial elastography resolution [40]. Stefano et al. obtained depth-dependent corneal displacements in live humans using swept-source OCE [41].

In this study, we developed an acoustic radiation force-based OCE system combined with an antisymmetric Lamb wave model to enable depth-resolved quantification of corneal biomechanics in vivo. Young’s modulus of the corneal cap and RSB were calculated, and the effect of the amount of stromal ablation in the SMILE surgery on the corneal biomechanical properties was analyzed.

## 2. Materials and Methods

### 2.1. Subjects

Twelve healthy adult New Zealand rabbits (weight: 3.5–4.5 kg, age: 6–7 m) were used in the refractive surgery. Unilateral eyes of these rabbits were randomly selected for the SMILE surgery and divided into two groups; Group Ⅰ: correction of −3.00 diopters (D), and Group Ⅱ: correction of −6.00 diopters (D) (*n* = 6/group). In the control group, a total of six healthy New Zealand White rabbits were selected and randomly chosen to complete the OCE experiment without any surgical treatment. The SMILE procedures and examinations were performed under anesthesia with an intramuscular injection of ketamine hydrochloride (20 mg/kg; Gutian Pharmaceuticals Co. Ltd., Fuzhou, China) and topical anesthesia with 0.4% Oxybuprocaine (Benoxil; Santen, Osaka, Japan). Subsequently, we euthanized the rabbits with an excess pentobarbital injection. All animal experiments and procedures were approved by the Ethics Committee of Nanchang Ophthalmic Hospital (Zhongshan Ophthalmic Center, Sun Yat-sen University), and the requirements of Association for Research in Vision and Ophthalmology Statement for the Use of Animals in Ophthalmic and Vision Research were fulfilled.

### 2.2. SMILE Procedure

The third eyelid of each rabbit was cut off before the procedure to completely expose the surgical area. The SMILE procedures were performed by the same surgeon using a 500 kHz femtosecond laser platform (Visumax; Carl Zeiss Meditec, Jena, Germany). In all cases, the surgery parameters were spot energy < 200 NJ, cap thickness = 110 µm, cap diameter = 7 mm, lenticule diameter = 6 mm, and refractive correction of -3D/-6D. The spot and track distances for the cap and lenticule were set at 4.3 µm, and the side cut was set at 2 µm. After surgery, all rabbits were treated with 0.5% levofloxacin (Santen, Osaka, Japan) four times a day for one week combined with 0.1% fluorometholone (Santen, Osaka, Japan) four times a day for one month to prevent infection.

### 2.3. Pre- and Postoperative Examinations

A slit-lamp microscope (TOPCON, Tokyo, Japan) was routinely used to exclude pre-operative pathological problems, and post-operative corneal healing was observed. Central corneal thickness (CCT) was obtained using ultrasound pachymetry (Tomey Corp, Nagoya, Japan). After the refractive surgery, the OCE system was used for imaging detection in the corneal tissue to obtain corneal elasticity parameters. The above examinations were completed 10 days before and 30 days after the surgery, respectively.

### 2.4. OCE System Setup

**Figure 1:**
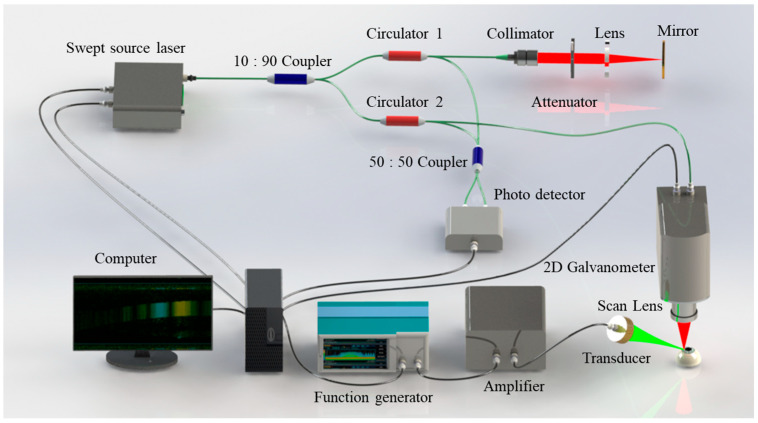
The schematic of the corneal OCE system setup.

For corneal elastography, the OCE system was composed of a home-built swept source OCT (SS-OCT) system and an acoustic radiation force exciter, as shown in Figure 1. In summary, the SS-OCT system employed a swept laser source with a central wavelength of 1310 nm, a wavelength scan range of approximately 95 nm, an A-line rate of 50 kHz, and output power of approximately 10 mW. The parameters of the OCE system had an axial resolution of 10 µm, a lateral resolution of 15 µm, a signal to noise (SNR) of 101 dB, and a displacement sensitivity of approximately 20 nm in the corneal experiments. The acoustic radiation force exciter consisted of a customized focused ultrasound transducer, a bandwidth power amplifier, and a function generator. Both the axial and the lateral range of the focal field were 1 mm. The maximum acoustic intensity in the cornea was 9 mW/cm^2^, which was less than the limit of 17 mW/cm^2^ for clinical ophthalmic ultrasound [42]. The M–B mode program was developed by synchronizing the ultrasonic pulse with the SS-OCT trigger to map and detect the elastic wave. In particular, a total of 500 M-scans were repeated for each A-line on the cornea to obtain the axial vibration information, and a total of 1000 A-line positions were acquired to form a B-scan. Because of the A-line acquisition speed, the temporal resolution in the OCE M–B mode imaging was 0.02 ms. During the M-scan of each A-line position, the ultrasound transducer was triggered 20 times—0.4 ms in total—to complete the mechanical excitation.

### 2.5. Antisymmetric Lamb Wave Model for Cornea

The cornea tissue is a nonlinear viscoelastic biological medium. Moreover, characterization of the corneal biomechanics of the cornea tissue using the shear wave group velocity is difficult, because the mechanical wave propagation is dispersive and depends on the frequency in the corneal tissue. In this study, we developed an antisymmetric Lamb wave model to quantify the biomechanical properties of the cornea with a high accuracy [43,44]. The antisymmetric Lamb wave dispersion equation can be expressed as [45,46]:

(1) $$4k_{L}^{3}\beta_{L}\cosh\left(k_{L}h\right)\sinh\left(\beta_{L}h\right)-{\left(k_{s}^{2}-2k_{L}^{2}\right)}^{2}\times \;\begin{array}{c} \sin h\left(k_{L}h\right)\cosh\left(\beta_{L}h\right)=k_{s}^{4}\cosh\left(k_{L}h\right)\cosh\left(\beta_{L}h\right), \end{array}$$

where $\beta_{L}=\sqrt{k_{L}^{2}-k_{s}^{2}}$, $k_{L}=\frac{2\pi f}{c_{L}}$ is the wave-number of the Lamb wave, *f* is the frequency of the Lamb wave, and $c_{L}$ is the Lamb wave phase velocity with frequency. In addition, $k_{s}=2\pi f\sqrt{\rho_{m}/U}$ is the wave-number of the shear wave; $\rho_{m}$ is the density of the tissue; *h* is the half-thickness of the cornea; and *U* is the represented viscoelastic of the tissue with U=μ+iωη in the Kelvin–Voigt model, where μ is the elasticity and η is the viscosity. Moreover, η can be calculated by fitting to the frequency-dispersion curve of the phase velocity. In the post-processing part, all experimental data were processed using the MATLAB software R2021b (The MathWorks, Natick, MA, USA). The phase-resolved Doppler algorithm was used to detect the axial vibration displacement of Lamb waves [25]. The frequency-dependent dispersion curve of the Lamb wave in the wave-number domain can be obtained by a two-dimensional (2D) Fourier transform of the spatial-temporal displacement diagram. The wave-number $k_{L}$ for each frequency *f* in the wave-number space was obtained by calculating the maximum intensity at that frequency. The phase velocity $c_{L}$ was calculated as follows, Equation (2) [44]:

(2) $$\begin{array}{c} c_{L}=\frac{2\pi f}{k_{L}}, \end{array}$$

Furthermore, the cornea has five layers: epithelium, Bowman’s membrane, stroma, Descemet’s membrane, and endothelium. According to the described data processing method, the same curvature as the corneal surface was selected along the depth direction to quantify the phase velocity of Lamb wave propagation; thus, depth-resolved elastography could be realized [47]. Finally, Young’s modulus of the cornea was calculated as follows in Equation (3):

(3) $$\begin{array}{c} E=\frac{9\rho \times c_{L}^{4}}{{\left(2\pi \times f\times h\right)}^{2}}, \end{array}$$

### 2.6. Statistical Analysis

In this study, statistical analysis of all experimental results was completed using the R version 3.5.3 software. Additionally, a *t*-test was used to compare the differences in biomechanical properties of the corneal cap and RSB after -3D and -6D surgeries. In addition, the effect of the SMILE surgery with different amounts of stromal ablation on the corneal biomechanical properties was analyzed. All data were presented as mean ± SD, and the *p*-value less than 0.05 was considered significant statistically.

## 3. Results

### 3.1. OCE Examination before the SMILE Surgery

**Figure 2:**
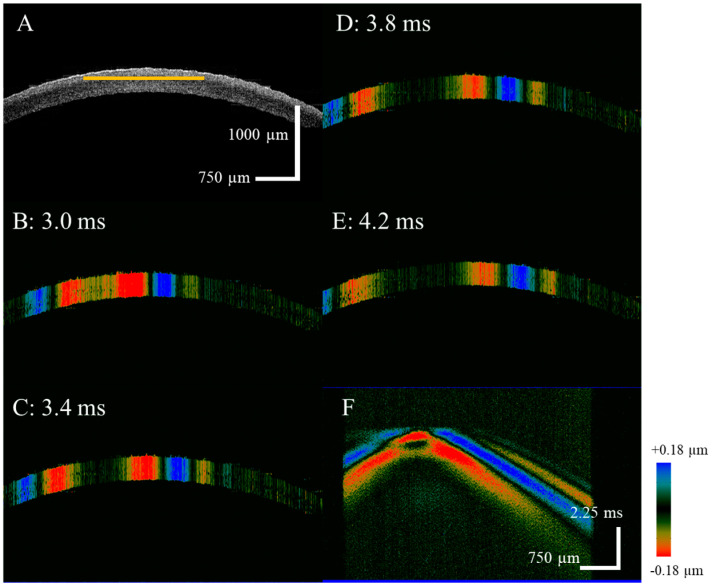
The OCE experiment results of the normal cornea, (**A**) is the corneal 2D structure image, (**B**–**E**) is the axial vibration with different times, and (**F**) is the spatial-temporal displacement image.

In the control group, the distribution of biomechanical properties in the depth direction of the normal cornea, measured by the OCE system, was used to determine the baseline data. Figure 2 shows the OCE experimental results of the normal cornea. In addition, Figure 2A presents the 2D corneal structure with five layers. During the OCE experiments, the acoustic radiation force pulse excites the corneal tissue to cause vibration, and the Lamb wave propagation process at different detection times was obtained using the M–B scan program. According to the phase resolved Doppler algorithm, the vibrational displacement of the Lamb wave $D\left(t\right)$ is determined by optical phase signal $\varphi \left(t\right)$ as: $D\left(t\right)=\frac{\lambda_{0}}{4\pi n}\cdot \varphi \left(t\right)$, $\lambda_{0}$ is the central wavelength of the swept source laser, and *n* is the air refractive index, the results are shown in Figure 2B–E. The red and blue colors indicate different vibration displacement directions. Figure 2F shows the spatial-temporal displacement map of the cornea using a resliced M–B image along the depth direction; the depth direction is indicated by the yellow line in Figure 2A.

**Figure 3:**
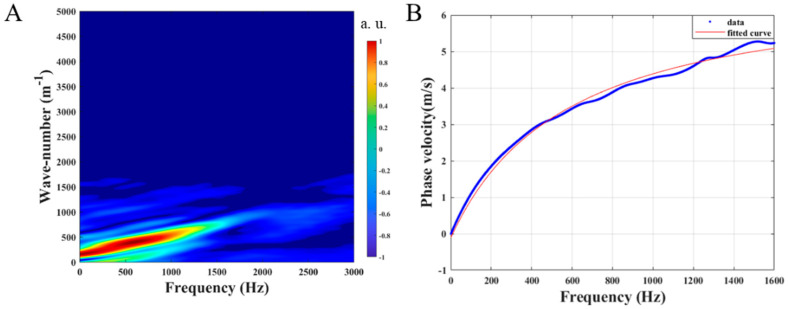
Phase velocity of the Lamb wave calculation, (**A**) is the wave-number frequency distribution image of the spatial-temporal displacement, and (**B**) is the phase velocity dispersion curve with frequency.

**Figure 4:**
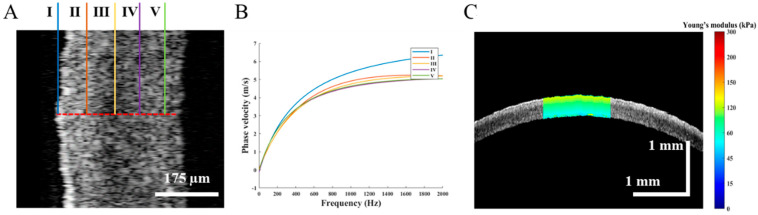
The different location Young’s modulus of the cornea, (**A**) is five different locations along the depth direction, (**B**) is the phase velocity curve of the Lamb wave at each location, and (**C**) is the depth-resolved Young’s modulus distribution of normal cornea.

Figure 3 presents the data post-processing for calculating the phase velocity of the Lamb wave depending on the dispersion frequency. The spectral distribution of the Lamb wave in the wave-number frequency domain is obtained using the 2D Fourier transform of the spatial-temporal displacement image, as shown in Figure 3A. The wave-number value corresponding to the maximum intensity is determined for each frequency. Subsequently, the phase velocity of the Lamb wave can be obtained by fitting the dispersion curve with the frequency, as expressed in Equation (2) and shown in Figure 3B. Furthermore, the phase velocity of the Lamb wave was calculated along the depth direction at five locations and different depths using the same procedure, each 70 µm apart, as illustrated in Figure 4A. The phase velocity of the Lamb wave gradually decreases, as shown in Figure 4B. As expressed in Equation (3), the depth-resolved of Young’s modulus of the normal cornea is quantified at each depth based on the same curvature of the anterior surface of the cornea. The normal corneal Young’s modulus gradually decreases from Bowman’s membrane to the endothelium in the range of approximately 112–79 kPa, as shown in Figure 4C.

### 3.2. Post-Operation OCE Examination of SMILE Surgery

Table 1 shows the values of pre- and post-operative CCT and RSB as measured using ultrasound pachymetry. Differences between the pre-operative CCT of the -3D and -6D groups (*p* = 0.09 > 0.05) were not significant. However, significant statistical differences between the post-operative CCT of the -3D and -6D groups appeared (*p* = 1.6 × 10^−5^ < 0.05). The thickness of RSB was calculated by subtracting the corneal cap thickness (−110 µm) from the post-operative CCT; a significant statistical difference between the two groups after the SMILE surgery appeared (*p* = 1.6 × 10^−5^ < 0.05).

**Figure 5:**
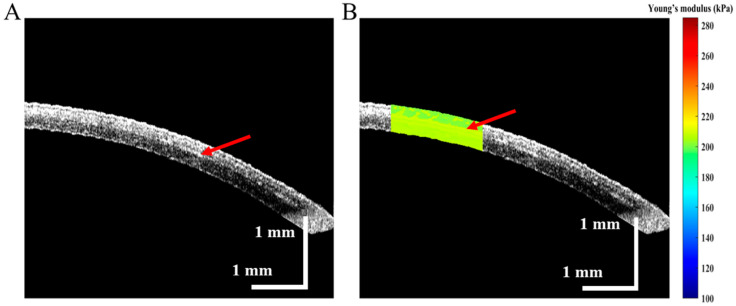
OCE results of cornea after -3D SMILE surgery, (**A**) is the 2D structure image and red arrow mark the boundary of the corneal cap and RSB, (**B**) is the depth-resolved Young’s modulus distribution of cornea, and the red arrow marks the distinct difference in Young’s modulus between the corneal cap and RSB.

In the -3D and -6D SMILE procedures, Young’s modulus of the corneal cap and RSB were quantitatively measured, and the post-operative stress distribution of the cornea was analyzed. During the data post-processing, a set of OCE experimental results was selected for detailed discussion. Young’s modulus was calculated three times and averaged to eliminate the corresponding measurement errors. Figure 5 demonstrates the OCE results of the -3D SMILE surgery group after the lenticule was removed. Moreover, the 2D structure of the cornea is illustrated in Figure 5A. The corneal cap and RSB were fused together using a negative pressure; the red arrow indicates the boundary. The biomechanical properties of the corneal cap and RSB for the -3D SMILE surgery group were obtained using the same method, as shown in Figure 5B. The average Young’s modulus of the corneal cap and RSB were 167.3 ± 3.2 and 179.0 ± 2.6 kPa, respectively. A distinct difference in the value of Young’s modulus between the corneal cap and RSB exists, as indicated by the red arrow in Figure 5B.

**Figure 6:**
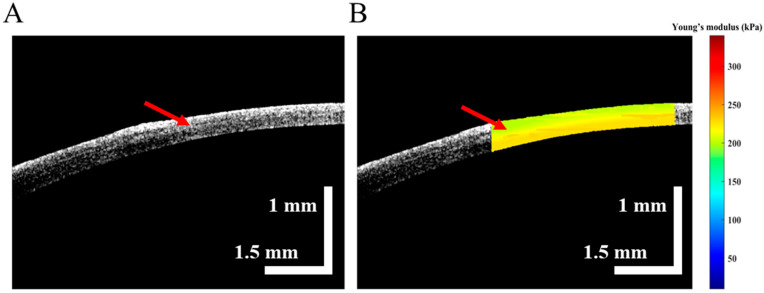
OCE results of cornea after -6D SMILE surgery, (**A**) is the 2D structure image and red arrow mark the boundary of the corneal cap and RSB, (**B**) is the depth-resolved Young’s modulus distribution of cornea, and the red arrow mark the distinct difference in Young’s modulus between the corneal cap and RSB.

The 2D corneal structure image of the -6D SMILE surgery is shown in Figure 6A, where the red arrow indicates the demarcation of the corneal cap and RSB. The biomechanical properties of the corneal cap and RSB was obtained, as shown in Figure 6B. Indeed, the average Young’s modulus of the corneal cap and RSB were about 213 ± 3.2 and 225 ± 4.0 kPa, respectively. In Figure 6B, the boundary of Young’s modulus changes is indicated by the red arrow; Young’s modulus of the corneal cap is lower than that of RSB.

**Figure 7:**
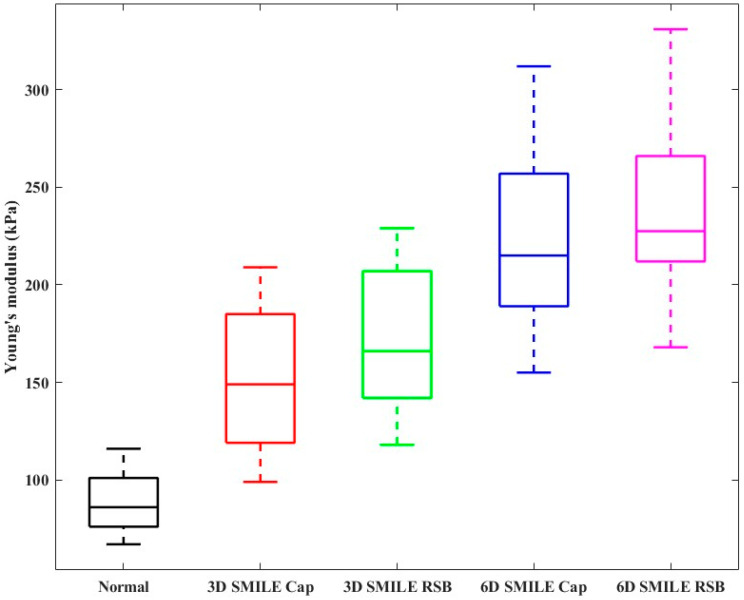
Statistical results of corneal Young’s modulus before SMILE and postoperative Young’s modulus of corneal cap and RSB in 3D and 6D group.

Furthermore, the results of the corneal Young’s modulus of the two groups before and after the SMILE surgery are shown in Figure 7. The average Young’s modulus of the normal corneal tissue was 88.7 ± 17.9 kPa in the control group. The average Young’s modulus of the corneal cap and the RSB for the -3D surgery group were 151.7 ± 42.2 kPa and 171.3 ± 41.7 kPa, respectively. In addition, the average Young’s modulus of corneal cap and RSB for the -6D surgery were 223.8 ± 54.8 kPa and 238.7 ± 55.2 kPa, respectively. Moreover, Young’s modulus of the -3D corneal cap is significantly lower than that of the -6D corneal cap (*p* = 0.0285 < 0.05), and Young’s modulus of the -3D RSB is significantly lower than that of the -6D RSB (*p* = 0.0384 < 0.05).

## 4. Discussion

In this study, we developed an acoustic radiation force OCE system to image and characterize the biomechanical properties of the in vivo cornea with a high spatial resolution and sensitivity. The corneal vibration induced by the acoustic radiation force was detected, which was used to quantify the corneal biomechanics based on a direct relationship between the phase velocity of Lamb waves and Young’s modulus. Consequently, the 2D Fourier transform of the spatiotemporal image of Lamb waves at each depth location was used to obtain the frequency-dependent phase velocity curve, subsequently. Young’s modulus of the corneal cap and the RSB after SMILE were calculated.

Corneal ectasia caused by iatrogenic factors is one of the serious complications of laser keratomileusis. Progressive thinning of the cornea leads to irreversible loss of vision. The causes of iatrogenic corneal ectasia are abnormal pre-operative biomechanical properties of the cornea and impaired biomechanics owing to the tissue removal [48]. Therefore, corneal biomechanical measurements are essential for pre-operative screening, surgical design, and post-operative monitoring [49]. Clinical testing instruments, including ocular response analyzers (ORA, Reichert, Buffalo, NY) and Corvis ST (Oculus, Wetzlar, Germany), obtain corneal biomechanical parameters by recording the deformation process of the cornea [50,51]. This method has a low sensitivity and cannot detect local differences in the corneal biomechanical properties, which are critical for refractive surgery [52]. OCE is an extension of the OCT technique that provides structural information in addition to Young’s modulus. In this study, we performed a high-resolution depth-resolved quantitative calculation of Young’s modulus of the cornea with an axial resolution of 10 µm, a lateral resolution of 15 µm, and a displacement sensitivity on the order of sub-nanometers. Thus, the effect of different parameters of the SMILE procedure on the corneal biomechanics could be analyzed.

As a soft tissue, the cornea exhibits a nonlinear mechanical behavior, and the stress–strain curve presents a typical “J” shape [53]. The cornea Young’s modulus can be expressed as the slope of its curve. Young’s modulus of the cornea increases with an increasing stress [54]. The relationship between the corneal Young’s modulus and IOP in in vivo normal human eyes was obtained using the ultrasonic surface wave elastography. The results showed that Young’s modulus increased with IOP; thus, changes in Young’s modulus characterized the distribution of stress [55]. The reported results were consistent with our results, which demonstrated that Young’s modulus of the cornea is increased after SMILE, as shown in Figure 7 SMILE disrupts the original weight-bearing structure of the cornea and increases the stress per unit area of the central cornea under IOP, resulting in a redistribution of stress to achieve a new equilibrium. Furthermore, the elasticity modulus of the corneal cap was elevated, indicating that the cap is still supporting the remaining cornea and able to tolerate the mechanical stress after SMILE. The tensile strength of the anterior corneal stroma is considerably higher than that of the posterior stroma [56]. In theory, SMILE has the advantage of preserving corneal biomechanics compared to flap-based procedures because it preserves most of the anterior corneal stroma; in addition, it is less damaging to the corneal biomechanics. Although Refs. [5,7] studies support the benefits of the SMILE procedure using a finite element model, clinical findings have not proved significant differences between SMILE and other procedures [9,57,58]. However, the results of these OCE experiments demonstrated that the corneal cap and RSB share IOP after SMILE, which contributes to the biomechanical stability of the post-operative cornea.

The amount of ablation of the corneal stroma is positively correlated with the magnitude of visual acuity corrected by refractive surgery. Excessive tissue ablation is a key cause of iatrogenic corneal ectasia after refractive surgery. Many indicators have been clinically proposed to assess this risk. A safe residual thickness of the post-operative stromal bed of greater than 250 μm is the current clinical consensus. In addition, the percentage of tissue change is used as a risk factor for predicting post-operative corneal ectasia [59,60]. In addition, the Ectasia risk scoring system serves as a valuable semi-quantitative screening method that integrates many risk indicators into a simple scoring system for pre-operative screening [4]. However, none of these methods can completely avoid the occurrence of iatrogenic keratitis. Another key factor in iatrogenic keratitis is weakened corneal biomechanics to a point where the corneal shape cannot be maintained [61]. An existing study proposed a mathematical calculation model to assess differences in the stromal tensile strength between different refractive surgery protocols; that study found that after removal of a cap of 130 μm and stromal tissue of 110 μm, post-operative corneal stromal tensile strength decreased by 25% [5]. Spiru et al. used a 2D stress–strain gauge to assess the biomechanical stability of ex vivo porcine cornea after SMILE [62]. At a strain of 0.8%, they observed a reduction of 2.5% in the post-operative corneal compression resistance. Our results prove that Young’s modulus of the -6D surgery group is significantly higher than that of the -3D surgery group after SMILE. The thinner the tissue in the corneal optic zone after surgery, the greater the stress on the unit tissue. The cornea reduced the local stress by increasing the strain (curvature). If considerable amount of tissue is removed or the pre-operative cornea is weak, the increased strain is not sufficient to restore a new equilibrium and may lead to a corneal dilation [63]. Therefore, quantitative detection of corneal biomechanics can provide useful information to effectively avoid the occurrence of post-operative corneal ectasia.

The main limitation of this study is that the structural parameters of the cornea (curvature, thickness, etc.,) are changed after refractive surgery, which will lead to errors in quantifying the distribution of biomechanical properties of the cornea anisotropy using the Lamb wave model. In addition, during the OCE experiments, the heartbeat and involuntary eye shaking of live rabbits cause background noises, which affect the calculation of the Lamb wave frequency and deviates from the estimation of corneal Young’s modulus [39]. Furthermore, after SMILE surgery, it takes time for the biomechanical properties of cornea to stabilize, and we completed only one OCE experiment one month postoperatively. To more closely resemble the postoperative process in the clinic, multiple experiments should be performed to evaluate the cross-sectional changes in biomechanical properties of cornea at different time periods. In the future, we will expand this study to alleviate its limitations.

In conclusion, we developed an OCE system combined with a Lamb wave model for depth-resolved and localized quantification of the corneal Young’s modulus. The proposed system has the advantages of non-destruction, high spatial resolution, and high sensitivity. Additionally, the effect of SMILE stromal ablation amount on the corneal biomechanics was further analyzed. The experimental results showed that Young’s modulus of the corneal cap and RSB significantly increased after SMILE, which was positively correlated with the amount of stromal resection. Although the increase in Young’s modulus of the corneal cap was not as high as that of RSB, they shared stress under IOP, which may contribute to the stabilization of corneal biomechanics after SMILE. Therefore, developing an OCE system for quantitative spatial characterization of the corneal biomechanical properties can provide effective information for the safe ablation range of SMILE procedures.

## Figures and Tables

**Table 1:** Central corneal thickness before and after the SMILE surgery.

Data (Mean ± SD)	-3D (*n* = 6)	-6D (*n* = 6)	*p*-Value
CCT (Pre-), µm	339 ± 3.4	345 ± 6.3	0.09 > 0.05
CCT (Post-), µm	278 ± 6.0	249 ± 6.9	1.6 × 10^−5^ < 0.05
RSB, µm	168 ± 6.0	139 ± 6.9	1.6 × 10^−5^ < 0.05

CCT = central corneal thickness, Pre- = preoperative, Post = postoperative, SMILE = small incision lenticule extraction, SD = standard deviation.

## Data Availability

Data can be obtained by contacting the corresponding author.
